# 6-Methyl-*N*-(2-methyl­phen­yl)-3-phenyl-1,6-dihydro-1,2,4,5-tetra­zine-1-carbox­amide

**DOI:** 10.1107/S1600536808020199

**Published:** 2008-07-09

**Authors:** Feng Xu, Weixiao Hu

**Affiliations:** a College of Pharmaceutical Science, Zhejiang University of Technology, Hangzhou 310032, People’s Republic of China

## Abstract

In the title compound, C_17_H_16_N_5_O, the central tetra­zine ring adopts an unsymmetrical boat conformation with the two C atoms as flagpoles. This compound can be considered as having homoaromaticity. The crystal structure is stabilized by inter­molecular C—H⋯O inter­actions between a benzene H atom and the carbonyl O atom.

## Related literature

For related literature, see: Hu *et al.* (2004 ▶, 2005 ▶); Jennison *et al.* (1986 ▶); Sauer (1996 ▶); Stam *et al.* (1982 ▶); Xu *et al.* (2006 ▶).
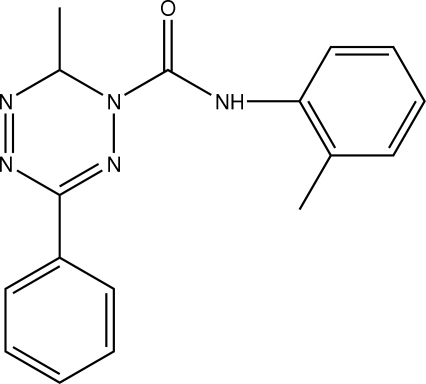


## Experimental

### 

#### Crystal data

C_17_H_16_N_5_O
*M* *_r_* = 306.35
Monoclinic, 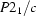

*a* = 13.941 (6) Å
*b* = 5.675 (2) Å
*c* = 20.614 (8) Å
β = 102.055 (6)°
*V* = 1594.9 (11) Å^3^
*Z* = 4
Mo *K*α radiation
μ = 0.08 mm^−1^
*T* = 291 (2) K
0.12 × 0.10 × 0.06 mm

#### Data collection

Bruker SMART APEX CCD area-detector diffractometer
Absorption correction: multi-scan (*SADABS*; Sheldrick, 1996 ▶) *T*_min_ = 0.980, *T*_max_ = 0.995
6815 measured reflections
3116 independent reflections
1882 reflections with *I* > 2σ(*I*)
*R*_int_ = 0.084

#### Refinement

*R*[*F* ^2^ > 2σ(*F* ^2^)] = 0.072
*wR*(*F* ^2^) = 0.193
*S* = 0.94
3116 reflections
226 parameters
H atoms treated by a mixture of independent and constrained refinement
Δρ_max_ = 0.54 e Å^−3^
Δρ_min_ = −0.31 e Å^−3^

### 

Data collection: *SMART* (Bruker, 2005 ▶); cell refinement: *SAINT* (Bruker, 2005 ▶); data reduction: *SAINT*; program(s) used to solve structure: *SHELXS97* (Sheldrick, 2008 ▶); program(s) used to refine structure: *SHELXL97* (Sheldrick, 2008 ▶); molecular graphics: *SHELXTL* (Sheldrick, 2008 ▶); software used to prepare material for publication: *SHELXTL*.

## Supplementary Material

Crystal structure: contains datablocks global, I. DOI: 10.1107/S1600536808020199/lx2060sup1.cif

Structure factors: contains datablocks I. DOI: 10.1107/S1600536808020199/lx2060Isup2.hkl

Additional supplementary materials:  crystallographic information; 3D view; checkCIF report

## Figures and Tables

**Table 1:** Hydrogen-bond geometry (Å, °)

*D*—H⋯*A*	*D*—H	H⋯*A*	*D*⋯*A*	*D*—H⋯*A*
C6—H6⋯O^i^	0.93	2.56	3.385 (3)	148

Symmetry code: (i) 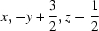.

**Fig. 1.**
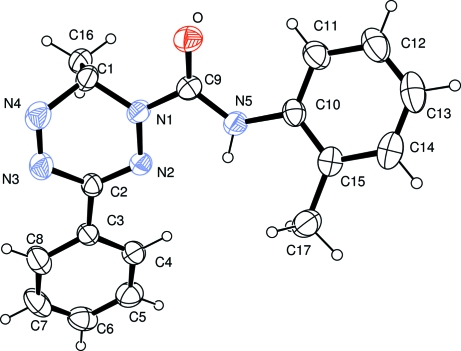
The structure of (I), shown with 30% probability displacement ellipsoids.

**Fig. 2.**
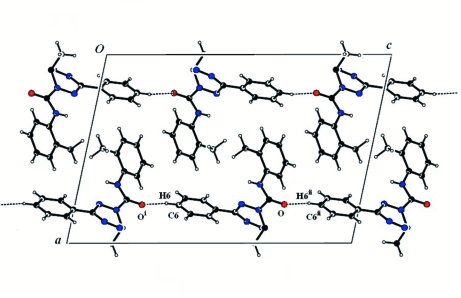
C—H···O interaction (dotted line) in the title compound. [Symmetry codes: (i) x, -y+3/2, z-1/2; (ii) x, -y+3/2, z+1/2.]

## supplementary crystallographic information

### Comment

1,2,4,5-Tetrazine derivatives have high potential for biological activity, possessing a wide spectrum of antiviral and antitumor properties. They have been widely used in pesticides and herbicides (Sauer, 1996). Dihydro-1,2,4,5-tetrazine has four isomers, namely 1,2-, 1,4-, 1,6-, and 3,6-dihydro-1,2,4,5-tetrazines. The 1,6-dihydro structures (Stam *et al.*, 1982; Jennison *et al.*, 1986) were found, by X-ray diffraction, to be homoaromatic. In continuation of our work on the structure–activity relationship of 1,6-dihydro-1,2,4,5-tetrazine derivatives (Hu *et al.*, 2004, 2005), we report the crystal structure of the title compound (I) (Fig. 1).

In the tetrazine ring, atoms N1, N2, N3 and N4 are coplanar, while atoms C1 and C2 deviate from the plane by 0.597 (3) and 0.225 (3)°, respectively. The N1/C1/N4 and N2/C3/N3 planes make dihedral angles of 42.3 (2)° and 19.7 (2)°, respectively, with the N1–N4 plane, *i.e.* the tetrazine ring adopts an unsymmetrical boat conformation. The C3–C8 benzene ring make dihedral angles of 13.2 (1)°, with the N1–N4 plane. N1 is almost *sp*^2^ hybridized due to the angles around it add up to 359.6 (2)°. In keeping with similar situations in 3-phenyl-6-ethyl-1,6-dihydro-1,2,4,5-tetrazine (Stam *et al.*, 1982), 3-(*p*-chlorophenyl)-6-methyl-1,6-dihydro-1,2,4,5-tetrazine (Xu *et al.*, 2006) and 1-acetyl-3,6-dimethyl-1,2,4,5-tetrazine (Jennison *et al.*, 1986), it can be considered that the molecule is homoaromatic.

The Fig. 2 shows that intramolecular C—H···O hydrogen bonds form a pseudo-five-membered ring. The crystal packing (Fig. 2) is stabilized by intermolecular C—H···O interactions  between a benzene H atom and the O atom of carbonyl group, with a C6—H6···O^i^ separation of 3.385 (3) Å (Table 1; symmetry code as in Fig. 2).

### Experimental

6-methyl-3-phenyl-1,6-dihydro-1,2,4,5-tetrazine (3.0 mmol), chloroform (10 ml) and pyridine (0.25 ml, 3.1 mmol) were mixed. 1-isocyanato-2-methylbenzene (3.0 mmol) in chloroform (10 ml) was added dropwise with stirring at room temperature. After the starting 1,6-dihydro-1,2,4,5-tetrazine was completely consumed (the reaction courses was monitored by TLC, dichloromethane system), evaporation of the chloroform, crude *N*-(*o*-methylphenyl) 3-phenyl-6-methyl-1,6-dihydro-1,2,4,5-tetrazine-1-carboxamide was obtained and purified by preparative thin-layer chromatography over silica gel PF254 (2 mm) (dichloromethane:petroleum ether = 1:1). The solution of the compound in anhydrous ethanol was concentrated gradually at room temperature to afford single crystals, which was suitable for X-ray diffraction. m.p. 378–380 K. Spectroscopic analysis: ^1^H NMR (CDCl_3_) δ p.p.m.: 8.64 (s, 1H), 8.14–8.16 (m, 2H, ArH), 7.92 (d, 1H, J = 8.0 Hz), 7.52–7.55 (m, 3H, ArH), 7.22 (m, 2*H*, ArH), 7.08 (t, 1H, J = 7.2 Hz), 6.91 (q, 1H, J=6.4 Hz), 2.34 (s, 3*H*), 1.09 (d, 3H, J = 6.8 Hz).

### Refinement

The positions of H atoms bound to C17 and N5 were obtained from difference Fourier map and refined isotropically. Other H atoms were placed in calculated positions with C—H = 0.93 (aromatic) and 0.96 Å (methyl), and refined in riding model, with *U*_iso_(H) = 1.5*U*_eq_(C)for methyl and 1.2*U*_eq_ for aromatic H atoms.

### Crystal data

**Table:**

C_17_H_16_N_5_O	*F*_000_ = 644
*M**_r_* = 306.35	*D*_x_ = 1.276 Mg m^−^^3^
Monoclinic, *P*2_1_/*c*	Melting point = 378–380 K
Hall symbol: -P 2ybc	Mo *K*α radiation λ = 0.71073 Å
*a* = 13.941 (6) Å	Cell parameters from 742 reflections
*b* = 5.675 (2) Å	θ = 3.2–24.8º
*c* = 20.614 (8) Å	µ = 0.08 mm^−^^1^
β = 102.055 (6)º	*T* = 291 (2) K
*V* = 1594.9 (11) Å^3^	Prism, red
*Z* = 4	0.12 × 0.10 × 0.06 mm

### Data collection

**Table:**

Bruker SMART APEX CCD area-detector diffractometer	3116 independent reflections
Radiation source: fine-focus sealed tube	1882 reflections with *I* > 2σ(*I*)
Monochromator: graphite	*R*_int_ = 0.084
Detector resolution: 10.0 pixels mm^-1^	θ_max_ = 26.0º
*T* = 293(2) K	θ_min_ = 1.5º
φ and ω scans	*h* = −17→8
Absorption correction: multi-scan(SADABS; Sheldrick, 1996)	*k* = −6→7
*T*_min_ = 0.980, *T*_max_ = 0.995	*l* = −25→25
6815 measured reflections	

### Refinement

**Table:**

Refinement on *F*^2^	Hydrogen site location: inferred from neighbouring sites
Least-squares matrix: full	H atoms treated by a mixture of independent and constrained refinement
*R*[*F*^2^ > 2σ(*F*^2^)] = 0.072	*w* = 1/[σ^2^(*F*_o_^2^) + (0.1133*P*)^2^] where *P* = (*F*_o_^2^ + 2*F*_c_^2^)/3
*wR*(*F*^2^) = 0.193	(Δ/σ)_max_ < 0.001
*S* = 0.94	Δρ_max_ = 0.54 e Å^−^^3^
3116 reflections	Δρ_min_ = −0.31 e Å^−^^3^
226 parameters	Extinction correction: SHELXL97 (Sheldrick, 2008), Fc^*^=kFc[1+0.001xFc^2^λ^3^/sin(2θ)]^-1/4^
Primary atom site location: structure-invariant direct methods	Extinction coefficient: 0.031 (5)
Secondary atom site location: difference Fourier map	

### Special details

**Table:**

Geometry. All esds (except the esd in the dihedral angle between two l.s. planes) are estimated using the full covariance matrix. The cell esds are taken into account individually in the estimation of esds in distances, angles and torsion angles; correlations between esds in cell parameters are only used when they are defined by crystal symmetry. An approximate (isotropic) treatment of cell esds is used for estimating esds involving l.s. planes.
Refinement. Refinement of F^2^ against ALL reflections. The weighted R-factor wR and goodness of fit S are based on F^2^, conventional R-factors R are based on F, with F set to zero for negative F^2^. The threshold expression of F^2^ > 2sigma(F^2^) is used only for calculating R-factors(gt) etc. and is not relevant to the choice of reflections for refinement. R-factors based on F^2^ are statistically about twice as large as those based on F, and R- factors based on ALL data will be even larger.

### Fractional atomic coordinates and isotropic or equivalent isotropic displacement parameters (Å^2^)

**Table:**

	*x*	*y*	*z*	*U*_iso_*/*U*_eq_	
O	0.79549 (13)	0.2358 (3)	0.73526 (8)	0.0685 (5)	
N1	0.82379 (14)	0.4541 (3)	0.64939 (9)	0.0552 (5)	
N2	0.79315 (13)	0.5181 (3)	0.58485 (9)	0.0522 (5)	
N3	0.88534 (17)	0.8632 (4)	0.61618 (12)	0.0685 (6)	
N4	0.91853 (17)	0.7948 (4)	0.67409 (12)	0.0724 (6)	
N5	0.68918 (15)	0.2115 (4)	0.63448 (10)	0.0598 (6)	
H5N	0.6759 (17)	0.282 (4)	0.5967 (12)	0.054 (6)*	
C1	0.91906 (18)	0.5354 (4)	0.68427 (11)	0.0587 (6)	
C2	0.83633 (17)	0.7032 (4)	0.56768 (11)	0.0526 (6)	
C3	0.82173 (17)	0.7755 (4)	0.49788 (12)	0.0542 (6)	
C4	0.77371 (19)	0.6256 (5)	0.44798 (12)	0.0644 (7)	
H4	0.7490	0.4824	0.4592	0.077*	
C5	0.7624 (2)	0.6873 (6)	0.38197 (14)	0.0813 (9)	
H5	0.7307	0.5853	0.3491	0.098*	
C6	0.7975 (2)	0.8971 (6)	0.36496 (16)	0.0825 (9)	
H6	0.7889	0.9392	0.3205	0.099*	
C7	0.8452 (3)	1.0452 (6)	0.41287 (18)	0.0876 (10)	
H7	0.8701	1.1870	0.4009	0.105*	
C8	0.8571 (2)	0.9862 (5)	0.47985 (15)	0.0788 (8)	
H8	0.8889	1.0897	0.5123	0.095*	
C9	0.76906 (18)	0.2904 (4)	0.67787 (11)	0.0535 (6)	
C10	0.61865 (18)	0.0506 (4)	0.64801 (11)	0.0559 (6)	
C11	0.6437 (2)	−0.1223 (5)	0.69669 (13)	0.0724 (8)	
H11	0.7077	−0.1329	0.7210	0.087*	
C12	0.5740 (3)	−0.2762 (6)	0.70856 (15)	0.0883 (10)	
H12	0.5905	−0.3910	0.7412	0.106*	
C13	0.4807 (3)	−0.2619 (6)	0.67292 (18)	0.0928 (11)	
H13	0.4330	−0.3646	0.6817	0.111*	
C14	0.4564 (2)	−0.0944 (6)	0.62345 (16)	0.0817 (9)	
H14	0.3926	−0.0891	0.5987	0.098*	
C15	0.52429 (19)	0.0652 (4)	0.60974 (12)	0.0601 (7)	
C16	1.00241 (18)	0.4238 (5)	0.65920 (13)	0.0658 (7)	
H16A	1.0013	0.2564	0.6656	0.099*	
H16B	1.0636	0.4865	0.6832	0.099*	
H16C	0.9955	0.4575	0.6128	0.099*	
C17	0.4969 (3)	0.2482 (6)	0.55605 (17)	0.0790 (8)	
H17A	0.431 (3)	0.232 (5)	0.5290 (16)	0.102 (10)*	
H17B	0.543 (2)	0.223 (5)	0.5212 (15)	0.092 (9)*	
H17C	0.511 (3)	0.408 (7)	0.5726 (16)	0.107 (11)*	

### Atomic displacement parameters (Å^2^)

**Table:**

	*U*^11^	*U*^22^	*U*^33^	*U*^12^	*U*^13^	*U*^23^
O	0.0755 (12)	0.0808 (13)	0.0475 (10)	−0.0050 (9)	0.0090 (8)	0.0014 (7)
N1	0.0491 (11)	0.0653 (12)	0.0486 (10)	−0.0056 (10)	0.0039 (8)	0.0001 (8)
N2	0.0469 (11)	0.0556 (11)	0.0529 (11)	−0.0018 (9)	0.0077 (8)	0.0026 (8)
N3	0.0682 (14)	0.0511 (12)	0.0823 (15)	−0.0009 (10)	0.0067 (12)	−0.0101 (10)
N4	0.0740 (15)	0.0636 (14)	0.0742 (15)	−0.0012 (11)	0.0030 (12)	−0.0181 (11)
N5	0.0537 (12)	0.0737 (14)	0.0501 (12)	−0.0109 (10)	0.0064 (10)	0.0102 (9)
C1	0.0521 (15)	0.0578 (14)	0.0616 (14)	−0.0068 (12)	0.0015 (11)	−0.0095 (10)
C2	0.0488 (13)	0.0424 (12)	0.0658 (14)	0.0002 (11)	0.0098 (11)	−0.0017 (9)
C3	0.0427 (13)	0.0485 (13)	0.0722 (15)	0.0022 (10)	0.0137 (11)	0.0085 (10)
C4	0.0610 (16)	0.0681 (16)	0.0637 (15)	−0.0068 (13)	0.0123 (12)	0.0116 (11)
C5	0.0763 (19)	0.101 (2)	0.0649 (16)	−0.0060 (17)	0.0117 (14)	0.0133 (14)
C6	0.0736 (19)	0.095 (2)	0.0832 (19)	0.0109 (18)	0.0271 (16)	0.0321 (17)
C7	0.094 (2)	0.0653 (19)	0.115 (3)	−0.0012 (17)	0.047 (2)	0.0308 (17)
C8	0.084 (2)	0.0583 (17)	0.098 (2)	−0.0070 (15)	0.0271 (16)	0.0097 (14)
C9	0.0520 (14)	0.0604 (14)	0.0484 (13)	0.0014 (11)	0.0111 (11)	−0.0033 (10)
C10	0.0597 (15)	0.0570 (14)	0.0548 (13)	−0.0092 (12)	0.0210 (11)	−0.0027 (10)
C11	0.0846 (19)	0.0703 (17)	0.0646 (15)	−0.0100 (15)	0.0209 (14)	0.0082 (12)
C12	0.119 (3)	0.080 (2)	0.0732 (18)	−0.028 (2)	0.035 (2)	0.0027 (14)
C13	0.106 (3)	0.089 (2)	0.097 (2)	−0.042 (2)	0.053 (2)	−0.0174 (18)
C14	0.0644 (18)	0.090 (2)	0.096 (2)	−0.0193 (16)	0.0277 (16)	−0.0261 (17)
C15	0.0571 (16)	0.0621 (15)	0.0645 (14)	−0.0049 (12)	0.0205 (12)	−0.0115 (11)
C16	0.0513 (15)	0.0635 (16)	0.0779 (16)	−0.0031 (12)	0.0029 (12)	0.0007 (11)
C17	0.064 (2)	0.078 (2)	0.087 (2)	0.0031 (17)	−0.0032 (17)	−0.0037 (16)

### Geometric parameters (Å, °)

**Table:**

O—C9	1.204 (3)	C7—C8	1.397 (4)
N1—N2	1.359 (3)	C7—H7	0.9300
N1—C9	1.406 (3)	C8—H8	0.9300
N1—C1	1.447 (3)	C10—C15	1.388 (4)
N2—C2	1.296 (3)	C10—C11	1.395 (4)
N3—N4	1.249 (3)	C11—C12	1.366 (4)
N3—C2	1.416 (3)	C11—H11	0.9300
N4—C1	1.487 (3)	C12—C13	1.356 (5)
N5—C9	1.351 (3)	C12—H12	0.9300
N5—C10	1.412 (3)	C13—C14	1.383 (5)
N5—H5N	0.86 (2)	C13—H13	0.9300
C1—C16	1.506 (3)	C14—C15	1.381 (4)
C2—C3	1.469 (3)	C14—H14	0.9300
C3—C8	1.374 (4)	C15—C17	1.508 (4)
C3—C4	1.394 (4)	C16—H16A	0.9600
C4—C5	1.382 (4)	C16—H16B	0.9600
C4—H4	0.9300	C16—H16C	0.9600
C5—C6	1.361 (4)	C17—H17A	0.98 (4)
C5—H5	0.9300	C17—H17B	1.07 (3)
C6—C7	1.360 (5)	C17—H17C	0.97 (4)
C6—H6	0.9300		
			
N2—N1—C9	119.9 (2)	O—C9—N5	127.3 (2)
N2—N1—C1	118.0 (2)	O—C9—N1	119.9 (2)
C9—N1—C1	121.7 (2)	N5—C9—N1	112.8 (2)
C2—N2—N1	114.5 (2)	C15—C10—C11	121.1 (2)
N4—N3—C2	120.2 (2)	C15—C10—N5	117.7 (2)
N3—N4—C1	115.6 (2)	C11—C10—N5	121.1 (2)
C9—N5—C10	126.5 (2)	C12—C11—C10	119.9 (3)
C9—N5—H5N	115.8 (16)	C12—C11—H11	120.1
C10—N5—H5N	117.0 (16)	C10—C11—H11	120.1
N1—C1—N4	105.6 (2)	C13—C12—C11	120.2 (3)
N1—C1—C16	112.9 (2)	C13—C12—H12	119.9
N4—C1—C16	110.4 (2)	C11—C12—H12	119.9
N2—C2—N3	120.7 (2)	C12—C13—C14	120.0 (3)
N2—C2—C3	121.1 (2)	C12—C13—H13	120.0
N3—C2—C3	117.4 (2)	C14—C13—H13	120.0
C8—C3—C4	118.4 (2)	C15—C14—C13	122.0 (3)
C8—C3—C2	121.6 (2)	C15—C14—H14	119.0
C4—C3—C2	120.0 (2)	C13—C14—H14	119.0
C5—C4—C3	120.7 (2)	C14—C15—C10	116.9 (2)
C5—C4—H4	119.7	C14—C15—C17	121.5 (3)
C3—C4—H4	119.7	C10—C15—C17	121.5 (2)
C6—C5—C4	120.2 (3)	C1—C16—H16A	109.5
C6—C5—H5	119.9	C1—C16—H16B	109.5
C4—C5—H5	119.9	H16A—C16—H16B	109.5
C7—C6—C5	120.1 (3)	C1—C16—H16C	109.5
C7—C6—H6	120.0	H16A—C16—H16C	109.5
C5—C6—H6	120.0	H16B—C16—H16C	109.5
C6—C7—C8	120.6 (3)	C15—C17—H17A	114.5 (19)
C6—C7—H7	119.7	C15—C17—H17B	107.6 (16)
C8—C7—H7	119.7	H17A—C17—H17B	104 (2)
C3—C8—C7	120.1 (3)	C15—C17—H17C	112.6 (19)
C3—C8—H8	120.0	H17A—C17—H17C	113 (3)
C7—C8—H8	120.0	H17B—C17—H17C	105 (3)
			
C9—N1—N2—C2	166.3 (2)	C4—C3—C8—C7	−0.5 (4)
C1—N1—N2—C2	−21.5 (3)	C2—C3—C8—C7	177.5 (3)
C2—N3—N4—C1	10.6 (3)	C6—C7—C8—C3	1.0 (5)
N2—N1—C1—N4	52.3 (3)	C10—N5—C9—O	1.3 (4)
C9—N1—C1—N4	−135.5 (2)	C10—N5—C9—N1	−178.6 (2)
N2—N1—C1—C16	−68.4 (3)	N2—N1—C9—O	−179.1 (2)
C9—N1—C1—C16	103.7 (2)	C1—N1—C9—O	8.9 (3)
N3—N4—C1—N1	−45.4 (3)	N2—N1—C9—N5	0.8 (3)
N3—N4—C1—C16	77.0 (3)	C1—N1—C9—N5	−171.2 (2)
N1—N2—C2—N3	−19.4 (3)	C9—N5—C10—C15	152.7 (2)
N1—N2—C2—C3	171.32 (19)	C9—N5—C10—C11	−29.4 (4)
N4—N3—C2—N2	25.8 (3)	C15—C10—C11—C12	−2.0 (4)
N4—N3—C2—C3	−164.6 (2)	N5—C10—C11—C12	−179.8 (2)
N2—C2—C3—C8	171.8 (2)	C10—C11—C12—C13	0.3 (4)
N3—C2—C3—C8	2.2 (3)	C11—C12—C13—C14	1.4 (5)
N2—C2—C3—C4	−10.2 (4)	C12—C13—C14—C15	−1.6 (5)
N3—C2—C3—C4	−179.8 (2)	C13—C14—C15—C10	0.0 (4)
C8—C3—C4—C5	0.3 (4)	C13—C14—C15—C17	−179.5 (3)
C2—C3—C4—C5	−177.7 (2)	C11—C10—C15—C14	1.8 (3)
C3—C4—C5—C6	−0.5 (4)	N5—C10—C15—C14	179.7 (2)
C4—C5—C6—C7	1.0 (5)	C11—C10—C15—C17	−178.7 (3)
C5—C6—C7—C8	−1.2 (5)	N5—C10—C15—C17	−0.8 (3)

### Hydrogen-bond geometry (Å, °)

**Table:**

*D*—H···*A*	*D*—H	H···*A*	*D*···*A*	*D*—H···*A*
C6—H6···O^i^	0.93	2.56	3.385 (3)	148

Symmetry codes: (i) *x*, −*y*+3/2, *z*−1/2.
