# 6-Methyl-*N*-(2-methyl­phen­yl)-3-phenyl-1,6-dihydro-1,2,4,5-tetra­zine-1-carbox­amide. Corrigendum

**DOI:** 10.1107/S160053680903013X

**Published:** 2010-01-09

**Authors:** Feng Xu, Weixiao Hu

**Affiliations:** a College of Pharmaceutical Science, Zhejiang University of Technology, Hangzhou 310032, People’s Republic of China

## Abstract

The formula of the title compound in the paper by Xu & Hu [*Acta Cryst.* (2008), E**64**, o1432] is corrected.

## Abstract 2

Corrigendum to *Acta Cryst.* (2008), E**64**, o1432.

In the paper by Xu & Hu [*Acta Cryst.* (2008), E**64**, o1432], the chemical formula is corrected and the structure has been rerefined to include a missing H atom. The *Crystal data*, *Data collection* and *Refinement* sections are updated together with the hydrogen-bond data.

## Experimental

### 

#### Crystal data

C_17_H_17_N_5_O
*M* *_r_* = 307.36
Monoclinic, 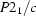

*a* = 13.941 (6) Å
*b* = 5.675 (2) Å
*c* = 20.614 (8) Å
β = 102.055 (6)°
*V* = 1594.8 (11) Å^3^
*Z* = 4
Mo *K*α radiation
μ = 0.08 mm^−1^
*T* = 293 (2) K
0.12 × 0.10 × 0.06 mm

#### Data collection

Bruker SMART APEX CCD area-detector diffractometer
Absorption correction: multi-scan (*SADABS*; Sheldrick, 1996 ▶) *T*_min_ = 0.990, *T*_max_ = 0.995
7095 measured reflections
3280 independent reflections
1899 reflections with *I* > 2σ(*I*)
*R*_int_ = 0.087

#### Refinement

*R*[*F* ^2^ > 2σ(*F* ^2^)] = 0.064
*wR*(*F* ^2^) = 0.170
*S* = 0.91
3280 reflections
215 parameters
1 restraint
H atoms treated by a mixture of independent and constrained refinement
Δρ_max_ = 0.54 e Å^−3^
Δρ_min_ = −0.31 e Å^−3^

## Supplementary Material

Crystal structure: contains datablocks global, I. DOI: 10.1107/S160053680903013X/lx9060sup1.cif

Structure factors: contains datablocks I. DOI: 10.1107/S160053680903013X/lx9060Isup2.hkl

## Figures and Tables

**Table 1:** Hydrogen-bond geometry (Å, °)

*D*—H⋯*A*	*D*—H	H⋯*A*	*D*⋯*A*	*D*—H⋯*A*
C6—H6⋯O^i^	0.93	2.56	3.385 (3)	148

Symmetry code: (i) 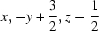.
